# Mirror-Induced Behavior in the Magpie (Pica pica): Evidence of Self-Recognition 

**DOI:** 10.1371/journal.pbio.0060202

**Published:** 2008-08-19

**Authors:** Helmut Prior, Ariane Schwarz, Onur Güntürkün

**Affiliations:** 1 Institut für Psychologie, Goethe-Universität, Frankfurt am Main, Germany; 2 Institut für Kognitive Neurowissenschaften, Biopsychologie, Ruhr-Universität Bochum, Bochum, Germany; Emory University, United States of America

## Abstract

Comparative studies suggest that at least some bird species have evolved mental skills similar to those found in humans and apes. This is indicated by feats such as tool use, episodic-like memory, and the ability to use one's own experience in predicting the behavior of conspecifics. It is, however, not yet clear whether these skills are accompanied by an understanding of the self. In apes, self-directed behavior in response to a mirror has been taken as evidence of self-recognition. We investigated mirror-induced behavior in the magpie, a songbird species from the crow family. As in apes, some individuals behaved in front of the mirror as if they were testing behavioral contingencies. When provided with a mark, magpies showed spontaneous mark-directed behavior. Our findings provide the first evidence of mirror self-recognition in a non-mammalian species. They suggest that essential components of human self-recognition have evolved independently in different vertebrate classes with a separate evolutionary history.

## Author Summary

A crucial step in the emergence of self-recognition is the understanding that one's own mirror reflection does not represent another individual but oneself. In nonhuman species and in children, the “mark test” has been used as an indicator of self-recognition. In these experiments, subjects are placed in front of a mirror and provided with a mark that cannot be seen directly but is visible in the mirror. Mirror self-recognition has been shown in apes and, recently, in dolphins and elephants. Although experimental evidence in nonmammalian species has been lacking, some birds from the corvid family show skill in tasks that require perspective taking, a likely prerequisite for the occurrence of mirror self-recognition. Using the mark test, we obtained evidence for mirror self-recognition in the European Magpie, Pica pica. This finding shows that elaborate cognitive skills arose independently in corvids and primates, taxonomic groups with an evolutionary history that diverged about 300 million years ago. It further proves that the neocortex is not a prerequisite for self-recognition.

## Abstract 3

The European magpie, a bird with a large brain and highly developed perspective-taking skills, shows evidence of mirror self-recognition.

## Introduction

Since the pioneering work by Gallup [1], a number of studies have investigated the occurrence of mirror-induced self-directed behavior in animals of a great range of species. Most animals exposed to a mirror respond with social behavior, e.g., aggressive displays, and continue to do so during repeated testing. In a few ape species, however, behavior changes over repeated presentations with a mirror. Social behavior decreases, and the mirror is used for exploration of the own body. This suggestive evidence of self-recognition is further corroborated by the mirror and mark test. If an individual is experimentally provided with a mark that cannot be directly seen but is, however, visible in the mirror, increased exploration of the own body and self-directed actions towards the mark suggest that the mirror image is being perceived as self. Fairly clear evidence of this has been obtained for chimpanzees [1], orang-utans [2], and pygmy chimpanzees [3]. In gorillas and gibbons, some authors reported failure of self-recognition [4,5] whereas others reported positive findings in at least one individual [6,7]. It should be mentioned that even in the chimpanzee, the species most studied and with the most convincing findings, clear-cut evidence of self-recognition is not obtained in all individuals tested. Prevalence is about 75% in young adults and considerably less in young and aging individuals [8]. Findings suggestive of self-recognition in mammals other than apes have been reported for dolphins [9] and elephants [10]. In monkeys, nonprimate mammals, and in a number of bird species, exploration of the mirror and social displays were observed, but no hints at mirror-induced self-directed behavior have been obtained [5]. Does this mean a cognitive Rubicon with apes and a few other species with complex social behavior on one side and the rest of the animal kingdom on the other side? This might imply that animal self-recognition is restricted to mammals with large brains and highly evolved social cognition but absent from animals without a neocortex.

Within humans and apes, self-recognition might reflect a homologous trait, whereas findings in other mammals hint at a convergent evolution. A likely reason for such convergent evolution of self-recognition in dolphins and elephants is the convergent evolution of complex social understanding and empathetic behavior [10]. If self-recognition is linked to highly developed social understanding, some birds species, in particular from the corvid family, are likely candidates for self-recognition, too. A number of studies from the past years have demonstrated an elaborated understanding of social relations, in particular during competition for food. It has been shown that own experience in pilfering caches facilitates predicting similar behavior in others [11], and that magpies [12] and scrub jays [13] remember who of their conspecifics observed them during storing. Thus, food-storing birds might be particularly apt in empathy and perspective taking, which have been suggested to coevolve with mirror self-recognition [14].

An investigation of self-recognition in corvids is not only of interest regarding the convergent evolution of social intelligence, it is also valuable for an understanding of the general principles that govern cognitive evolution and their underlying neural mechanisms. Mammals and birds inherited the same brain components from their last common ancestor nearly 300 million years ago and have since then independently developed a relatively large forebrain pallium. However, both classes differ substantially with regard to the internal organization of their pallium, with birds lacking a laminated cortex but having developed an organization of clustered forebrain entities instead [15]. In some groups of birds and mammals, such as corvids and apes, respectively, brain to-body ratios are especially high [16], and these animals are able to generate the same complex cognitive skills [17]. This is indicated by feats such as tool use and tool manufacture [18,19], episodic-like memory [20], and the ability to use own experience in predicting the behavior of conspecifics [11]. Although it has been shown that some birds, e.g., Grey Parrots [21], use mirrors with skill in order to localize and discriminate objects, no experimental evidence of self-recognition has been obtained in birds so far.

In the present study, magpies were chosen for several reasons. They are food-storing corvids that compete with conspecifics for individually cached and memorized hoards. They thus live under ecological conditions that favor the evolution of social intelligence [12,17]. They achieve the highest level of Piagetian object permanence [22], which is also achieved by apes, but not by monkeys. In addition to showing social understanding during competition for food [12], magpies are curious and prone to approach new situations, making them ideally suited for an experiment that requires spontaneous interaction with a new and puzzling context.

Mirror behavior in animals goes through several stages. In all species tested so far, inspection of the mirror and social behavior has been observed. In species with mirror self-recognition, some of the individuals also show evidence of inspection of their own body and testing for behavioral contingencies after familiarization with the mirror. For example, they move back and forth in front of the mirror, and this might indicate that they check to which degree the mirror image is coupled to their own movement. Individuals achieving this stage often also pass the mirror and mark test.

**Figure 1:**
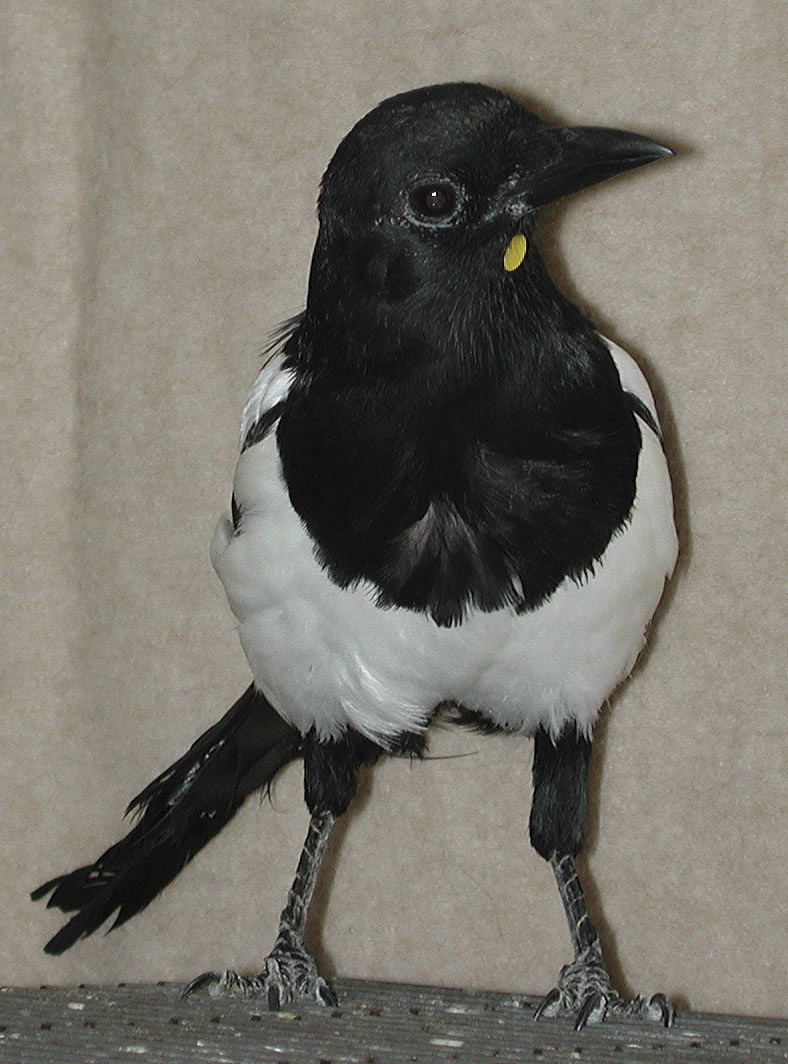
Magpie with Yellow Mark

**Figure 2:**
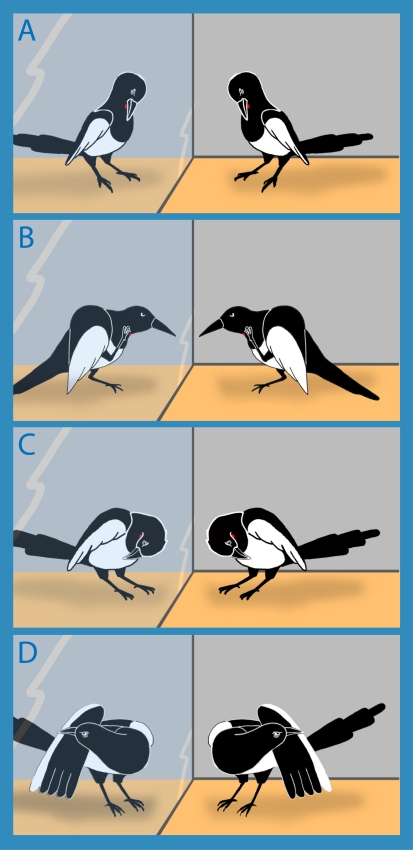
Examples of the Behaviors That Were Used for Quantitative Analysis (A) Attempt to reach the mark with the beak; (B) touching the mark area with the foot; (C) touching the breast region outside the marked area; (D) touching other parts of the body. Behaviors (A) and (B) entered the analysis as mark-directed behavior; behaviors (C) and (D) and similar actions towards other parts of the body were considered self-directed, but not related to the mark.

In our experiments, we began with open mirror exploration, and then we assessed preference for the mirror and quantified mirror-induced behaviors under highly standardized conditions in a two-compartment cage with one side containing a mirror. Subsequently, we investigated spontaneous self-directed behavior in individuals provided with a mark, and finally, we carried out a series of mark tests and control tests that were designed as to ensure appropriate control and exclude the possibility that findings are due to operant conditioning. Marked individuals (cf. Figure 1) were given a small number of tests, and we applied two types of appropriate controls. The birds were either marked with a brightly colored (yellow or red) or a black (sham) mark. Handling and somesthetic input was thus identical for all marks, but the black mark was practically not visible on the black feathers of the throat. In half of the trials, a mirror was placed with the reflective surface towards the animal; in the other half of trials, the mirror was replaced by a nonreflective plate of the same size and position. Therefore, the possibility to see a colored spot on the own body by means of the mirror was the only predictor of an increase of behavioral activities towards the marked (or sham-marked) region (Figure 2) in the different experimental conditions. Each bird was tested twice in each of the conditions, resulting in eight tests per bird.

## Results and Discussion

### Open Mirror Test

In baseline trials with a nonreflective plate, there was no remarkable behavior in front of the plate in any of the individuals. With a mirror, the behavior of the magpies clearly differed. Initial exploration of the mirror was characterized by approaches towards the mirror and looks behind the mirror. Also, social behavior occurred, such as aggressive displays towards the mirror and jumping towards the mirror as in a fight. In three individuals, Gerti, Goldie, and Schatzi, social behaviors were transient, i.e., they were reduced already on the second exposure or completely ceased to occur. In the other two birds, Harvey and Lilly, social behaviors, in particular aggressive and submissive displays, continued to be frequent. On several trials, Harvey also picked up little, but conspicuous, objects and posed, accompanied by wing-flipping, in front of the mirror holding the objects in the beak. This courtship-like behavior vanished after a few trials, and was never seen on later tests, which were characterized by aggressive displays. In the open mirror experiment, however, mainly two of the birds (Goldie and Harvey) took part, whereas the other three birds only occasionally visited the location of the mirror. Therefore, we proceeded with a highly standardized protocol for mirror exploration.

### Mirror Preference and Standardized Mirror Exploration

In these tests, birds could choose between two identical compartments of a cage, one equipped with a mirror and the other with a nonreflective plate instead of the mirror. Table 1 gives the time the birds spent with view on the mirror and shows how many bouts of close inspection of the mirror, of looks behind the mirror, of contingent behavior, and of social behavior were displayed by the birds. Three of the individuals (Gerti, Goldie, and Schatzi) spent a considerable amount of time in the compartment with the mirror, whereas the two other birds (Lilly and Harvey) appeared to avoid the compartment with the mirror. In the three birds with a preference for the mirror, behavior was characterized by close visual exploration of the mirror image (see Video S1 for an example). In addition, Gerti and Schatzi repeatedly looked behind the mirror and showed several bouts of behavior indicating contingency testing. Subjects moved their head or the whole body back and forth in front of the mirror in a systematic way (see Videos S2 and S3 for examples). In Goldie, contingent behavior was not demonstrated in this test, but demonstrated later in the mark test (see Video S4). Harvey and Lilly never showed any hint of such behavior. It is noteworthy that those birds that had a high interest in the mirror and also showed social displays only in the first tests were those that showed at least some evidence of self-directed behavior on later tests.

**Table 1:**
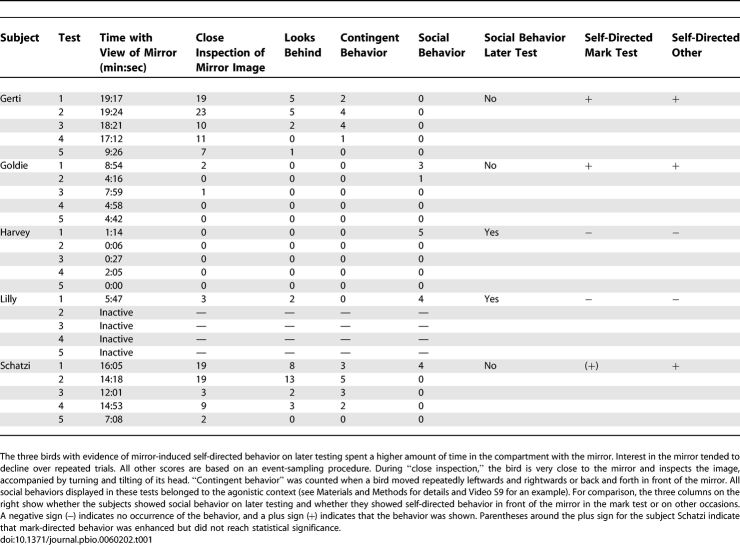
Behavioral Data from Mirror Preference and Standardized Mirror Exploration

### Mark Test

**Figure 3:**
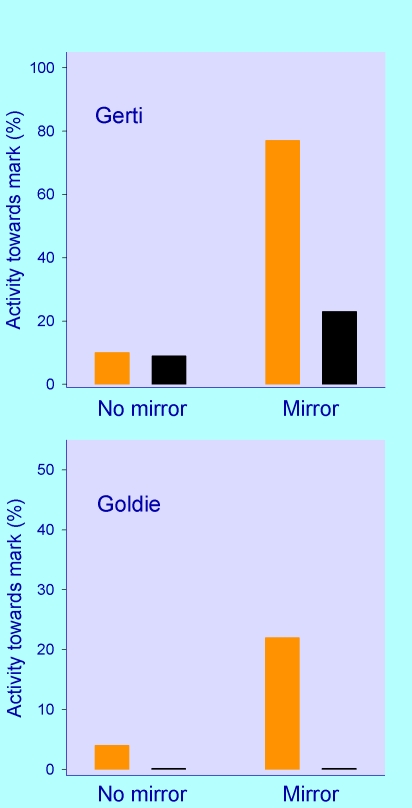
Proportion of Self-Directed Behavior towards the Mark Area Expressed as a Proportion of Overall Self-Directed Behavior in Subjects Gerti and Goldie Orange bars refer to tests with a colored mark (yellow or red), black bars to tests with a black control mark (sham condition). In Gerti (*p* < 0.005, Fisher exact test), as well as in Goldie (*p* < 0.05, Fisher exact test), mark-directed behavior was significantly enhanced in the colored mark and mirror condition.

**Figure 4:**
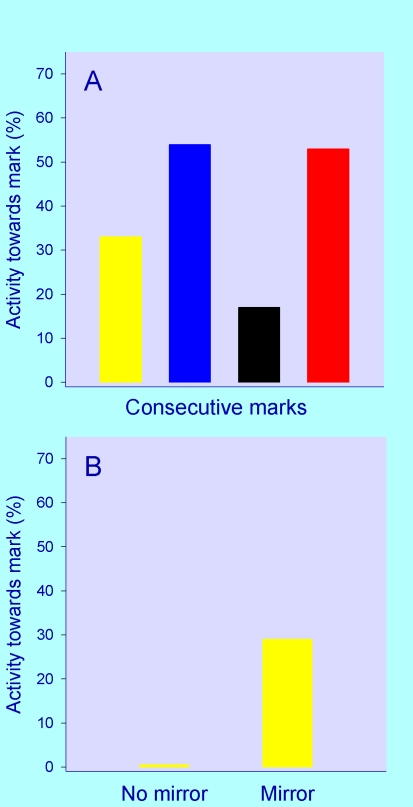
Proportion of Self-Directed Behavior towards the Mark Area in Additional Tests (A) Proportion of mark-directed behavior by Gerti in a test session with change of marks in consecutive 5-min intervals. The color of each bar refers to the color of the mark used and subsequent 5-min periods of the test. Activity towards the mark was high for all colored marks, but low in the control condition (black mark). In all of the color conditions, the bird removed the mark. (B) Results from the first set of additional controls with a grid in front of the experimental cage instead of a Plexiglas wall. In the colored mark and mirror condition, mark-directed behavior was higher than in the colored mark and no-mirror condition (*p* < 0.02, Fisher exact test).

In a first exposure to a mirror with a mark, three out of five birds showed at least one instance of spontaneous self-directed behavior (see Video S6 for an example in the subject Schatzi). In the subsequent quantitative analysis, which compared the behavior in the mirror and mark condition with a condition without a mirror, mark-directed behavior (cf. Videos S5 and S7) in two of the birds, Gerti and Goldie, was significantly higher in the critical mirror and mark condition than in the other conditions. There were no instances in any of the birds of pecking at the reflection of the mark in the mirror. Figure 3 shows the quantitative amount of behavior towards the mark region as a proportion of all behaviors towards the own body for these two birds. Contrary to the absolute counts, this proportion will only increase with a specific effect on mark-directed behavior but not as a consequence of a possible overall increase in behaviors towards the own body. It can be seen that mark-directed behavior was only significantly enhanced when a mirror was present and the mark was colored and thus visible for the birds. The detailed frequencies in the different conditions are given in Table 2. The comparison of the frequencies of behaviors directed towards other parts of the body clearly shows that the mark-directed behaviors in the mirror and colored mark condition cannot be explained by a general increase of behavioral activity. A specific increase of mark-directed behavior in presence of a mirror is corroborated by the fact that mark-directed behavior ceased within trials as soon as the bird had removed the mark (see Video S8). In Figure 4A, performance of Gerti in a single test with change of marks is shown (see also Table 3). As this is only one test, findings should be interpreted with some caution, but consistent with the between-sessions comparison in the first test series, mark-directed behavior was high when the mark was visible in the mirror and low with the black control mark that was not visible. Figure 4B shows the results of an additional test with two sessions in the mirror and colored mark (yellow) condition and two control sessions with colored marks and a nonreflective plate instead of the mirror. Also in this case, mark-directed behavior only occurred with the mirror. With colored mark and mirror, over the two trials, there were five mark-directed actions per trial and 12 actions towards the rest of the body, whereas there was no mark-directed behavior at all when the bird wore a colored mark but no mirror was present. Again, findings clearly show that this significant difference cannot be explained by an unspecific increase in overall behavioral activity, as the overall rate of behaviors directed towards the own body was similar. These quantitative data are rather conservative as Gerti and Goldie removed the mark after a few minutes on most of the trials with a color mark and a mirror, and after removal of the mark, no mark-directed behaviors occurred (see Videos S5, S7, and S8 for different aspects of mark-directed behavior in the subject Gerti).

**Table 2:**
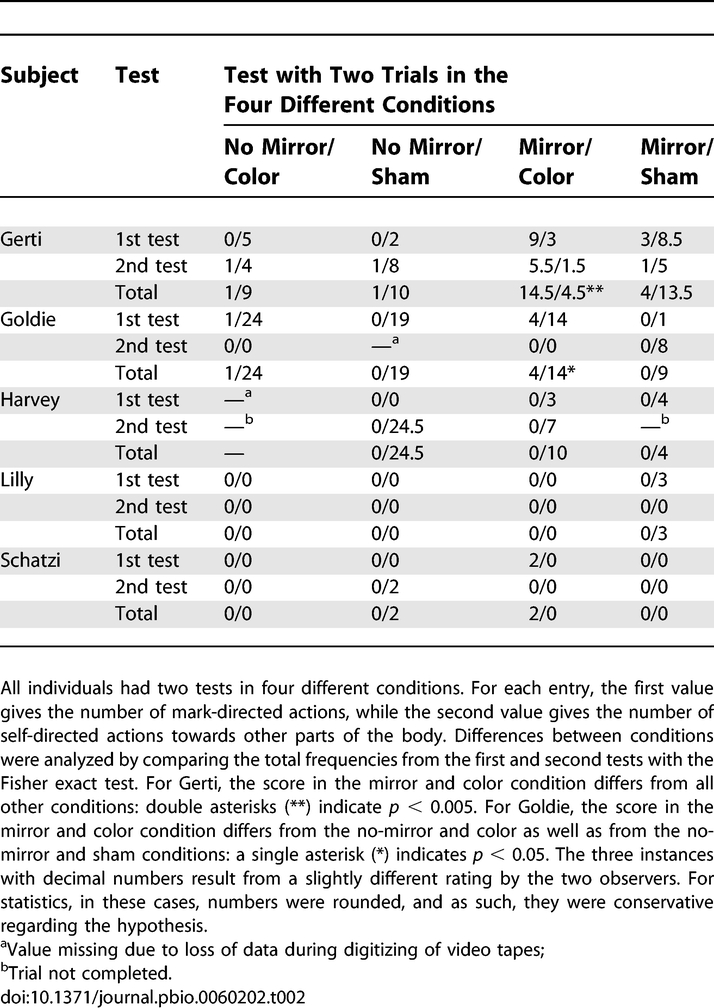
Frequencies of Self-Directed Behaviors in the Mark Test

**Table 3:**
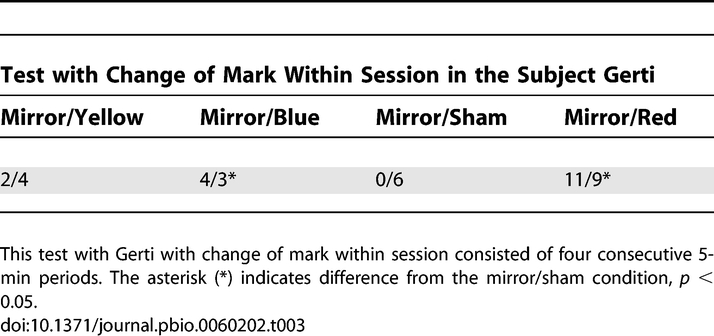
Frequencies of Self-Directed Behaviors in the Mark Test

Whereas mark-directed behaviors were virtually absent when the birds were tested without a mirror but with a colored mark, there were a few instances of mark-directed behavior in the mirror condition with a black control mark (Figure 3). It may well be that the black paper mark was slightly visible on the black plumage. This is supported by the observation that if the black mark condition elicited behavior, it was in the “mirror present” trials. This is an indirect support for the interpretation that the behavior towards the mark region was elicited by seeing the own body in the mirror in conjunction with an unusual spot on the own body.

Evidence from the quantitative data is corroborated by the qualitative behavior of the birds. Self-directed activity began after looks into the mirror and visual exploration of the mirror image, and it ceased as soon as the bird had successfully removed the mark (see Video S5). This is unlike chimpanzees, which, after discovering that the mark is inconsequential, rapidly lose interest [23]. The reason for the difference could be that bird's feathers are of considerably higher importance for survival than a patch of hair in chimps. This interpretation is supported by data showing that birds spend about one quarter of their resting time with preening and are often seen to interrupt sleeping in complete darkness only to preen [24].

Two of the other three birds reacted to the mirror with excited behavior characterized by frequent jumping and running within the cage, and the last bird showed a high number of attacks towards the mirror in one trial (see Video S9), but not in the other. The subject Schatzi, which had shown spontaneous mark-directed behavior during an earlier exposure, showed no significant mark-directed behavior in this series of tests, although there were two instances of mark-directed behavior in the mirror and color condition and no mark-directed behavior in the other conditions.

Interestingly, the behavior in the mark tests corresponded to interest in the mirror in the standardized mirror exploration test. Those individuals that showed at least one instance of mark-directed behavior were the same that had shown a high interest in the mirror in the preference test, and the individual strongly avoiding the mirror in the choice test (Harvey) showed a high amount of attack-like behavior in the mark test.

One might ask why rather clear evidence was observed in two individuals and weaker evidence in another one, but not in all of the five birds. The proportion of positive findings is, however, well in the range of what has been found in apes. In chimpanzees, the species best studied and showing the clearest evidence of mirror-induced self-directed behavior, a considerable number of individuals typically produce negative findings [8,25]. Of 92 individuals tested by Povinelli et al. [8], only 21 demonstrated clear and nine weak evidence of self-exploration, with about 75% prevalence in young adults of 8 to 15 y. Only half among those with clear evidence of self-exploration passed the mark test. Thus, our data do not only qualitatively, but also quantitatively, match the findings in chimpanzees. As a note of caution, we would like, however, to emphasize that the number of birds we tested is too small for a definitive estimate of the distribution within the population. Thus, further studies must assess whether the typical frequency of mirror-induced self-directed behavior in magpies is comparable to that in chimpanzees.

Altogether, results show that magpies are capable of understanding that a mirror image belongs to their own body. We do not claim that the findings demonstrate a level of self-consciousness or self-reflection typical of humans. The findings do however show that magpies respond in the mirror and mark test in a manner so far only clearly found in apes, and, at least suggestively, in dolphins and elephants. This is a remarkable capability that is at least a prerequisite of self-recognition and might play a role in perspective taking. It thus could be essential for the ability of using own experience to predict the behavior of conspecifics [11]. Magpies are corvids, which belong to the order of Passeriformes, a phylogenetic group characterized by large brains relative to body weight [26]. The relative brain size of passeriform birds is similar to primates in allometric analyses, and within the Passeriformes, corvids stand out with particular high relative brain size [27]. Thus, magpies belong to a group of animals with very high relative brain size (see also Table 4).

**Table 4:**
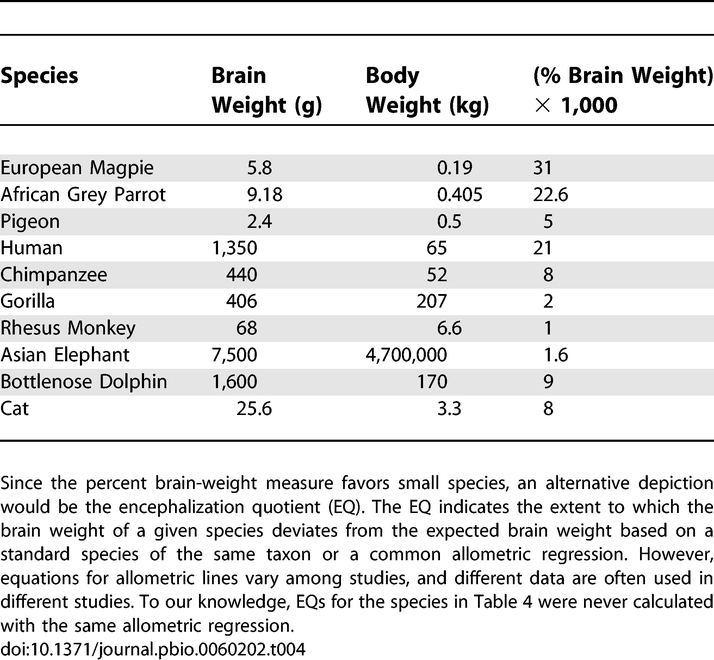
Brain Weights, Body Weights, as Well as Percent Brain-to-Body Weights of Some of the Different Species That Have Been Tested with Mirrors

We used a small number of tests as it was crucial to ensure that possible self-directed behavior of the birds represented a spontaneous response to seeing the own body in the mirror. Epstein et al. [28] reported that prolonged operant conditioning of isolated components of the mark test in pigeons could produce a behavioral pattern that superficially looks like mirror-induced mark-directed behavior. This study could, however, not be replicated [29], and these authors also found reduction of self-directed behavior in pigeons in the conditions with a mirror, which strikingly contrasts with our findings in magpies. Additionally, extremely long periods of exposure to mirrors without specific training of self-related actions did not produce any kinds of behavior that was centered on the mark in monkeys [30,31]. Lastly, the mark test is only one piece of evidence of mirror-induced self-recognition in animals. Of equal importance are previous inspections of the mirror, such as during looks at the back side of the mirror, and exploration of mirror properties, such as during contingent behavior [1,8,10,32]. Our magpies showed self-related behavior in front of the mirror after a rather short cumulative exposure time and without being specifically trained to do so. In addition, when confronted with mirrors the first time, they displayed similar sequences of behavior as described in apes [1,2,3,8,32]. Although the mark test has been criticized [33,34], the main objections have been ruled out [23], and it remains one of the most useful tests for self-recognition in comparative studies [35]. When magpies are judged by the same criteria as primates, they show self-recognition and are on our side of the “cognitive Rubicon.” One should keep in mind that though mirror self-recognition reflects a crucial step in the emergence of self-recognition, the fully fledged capacity is complex, and comparative [36,37], clinical [38], and developmental studies [39] suggest an overall gradual development of this capacity.

Cognitive and neurobiological studies of the last decade have shown that birds and mammals faced a similar selection pressure for complex cognitive abilities, resulting in the evolution of a comparable neural architecture of their forebrain association areas [40] as well as their cognitive operations [17,41–43]. This high degree of evolutionary convergence is especially visible for the cognitive abilities of corvids and apes [17]. By demonstrating self-recognition in the mirror by magpies, the present study shows that even the neural capacity for distinguishing self and others has evolved independently in the two vertebrate classes and that a laminated cortex is not a prerequisite for self-recognition.

## Materials and Methods

Five adult hand-raised magpies served as subjects throughout the study. These birds had been used before in a study on the development of object permanence and in patterned string problems. Investigation of behavior towards a mirror consisted of three steps.

### Open mirror exploration.

In a 4 × 4-m room, a mirror 55-cm wide and 40-cm high was placed on the ground, leaning against a pole, and slightly tilted. The position of the mirror was in the middle of one of the walls with about 1.5-m distance from the wall, allowing the birds to move around the mirror. The tested subject could move freely in the room. After a baseline trial with the mirror replaced by a grey, nonreflective plate, five test sessions of 30-min duration were given to each of the birds. The behavior of the birds was observed from an adjacent room by means of a video system, and trials were videotaped.

### Mirror preference and standardized mirror exploration.

For a quantitative estimation of the interest in the mirror, a cage with two opposite compartments was used. Compartments 60 × 100 × 60 cm (length × height × width) were identical except that there was a mirror at the end of one of the compartments and a grey, nonreflective plate of the same size in the other compartment. Between the compartments, there was a partition with two overlapping walls so that the birds could move freely between compartments but could not see from one compartment into the other. Each of the birds received five consecutive trials of 20 min on separate days. The time the birds spent in the compartment with the mirror was measured, and based of the videotapes bouts of close mirror inspection, looks behind the mirror, bouts of contingent behavior, and social behaviors were counted.

### Mark test.

In the mark test, each subject was involved in eight test sessions with the conditions, (1) mirror and colored mark, (2) mirror and black mark, (3) no mirror with colored mark, and (4) no mirror with black mark. One session lasted 20 min. Each condition was replicated once, and two colors, either yellow or red, were used for the colored mark. Thus, our subjects were marked in all conditions to prevent cueing by somesthetic input, but the black mark in condition 2 was practically not visible on the black feathers of the throat. Such sham marking, which also had been used in studies with dolphins and elephants [9,10], has the advantage that no anesthesia is needed, and it provides a rather rigorous control as except for the difference in appearance, every detail of the procedure is perfectly matched to the conditions with a color mark. In the no-mirror controls, the mirror was replaced by a nonreflective flat grey plate of the same size and in the same position. Conditions, including usage of the two marking colors yellow and red, alternated in balanced order. Colored marks and black control marks were fixed below the beak onto the throat region (Figure 1). Different pigeon breeds are blind to this area even during strong convergent eye movements [44]. In magpies, the visual field has not been studied in detail. However, the position of the eyes and the optical axis is comparable to that of pigeons. Thus, the assumption is justified that the spot used for marking was far outside the magpies' visual field. This is, furthermore, strongly corroborated by the behavioral data, as there was virtually no mark-directed behavior if magpies wore a colored mark in the no-mirror condition. In each of the conditions, every detail regarding the handling of the birds was identical, except that the birds could see the reflection of the yellow or red mark in the mirror and colored mark condition, but not in the various other conditions.

### Scoring of behaviors.

Before testing, we protocolled all behaviors observable. Then two observers (others than those assessing the mirror test) independently scored videotapes several times, and a list emerged of 18 behaviors with high interobserver agreement and high reliability over repeated scorings. The list included behaviors not relevant for the question of this study (like moving slowly or fast or jumping in the cage), and for the present study, we focused on a subset of behaviors diagnostic of the animals responding to the mirror. First, social behaviors that could be observed with high reliability were agonistic displays, either submissive or attack-like. During submissive behavior, the bird faces the mirror (or another bird), has its back lowered, its wings slightly spread, and often flips its wings. During aggressive displays, the bird takes an upright position with elongated neck and/or performs attack-like behaviors towards the mirror (c.f. [45] for pictures of typical displays by magpies). Secondly, we recorded all behaviors directed towards a bird's own body, such as touching any part of the body with the beak or the foot, and we assessed whether the action was directed towards the regions near the mark or elsewhere.

Before the first mark test, birds were familiarized with the experimental cage and the mirror for at least 5 d. The experimental cage was 120 × 100 × 60 cm (l × h × w) and had a grid floor. The walls also consisted of a grid except for one long wall, which in most of the tests consisted of Plexiglas in order to provide a good view of the bird. The mirror or the nonreflective plate was always placed on the ground at the same short wall of the cage.

At the beginning of a test, a bird was taken from its home cage and brought to an adjacent room, where the colored mark or the control mark was fixed. The bird was held by one of the experimenters such that the throat region below the beak was exposed. The head of the bird was shielded by the hand of the experimenter holding the bird so that the bird could not see the fixing procedure. The other experimenter then fixed the self-adhesive colored or black mark. Except for the color of the mark, the handling procedure was exactly the same in each of the experimental conditions. Although the dots used for marking were self-adhesive, we prepared them with double-sided adhesive tape in order to ensure good fixation. The weight of a dot was 16 μg and the diameter 8 mm.

After completion of testing of all birds in each of the conditions twice, further tests were applied for the subject Gerti, who showed very clear and consistent self-directed behavior. First of all, a mirror test with four consecutive phases of 5 min was carried out, and in each phase, a new mark was fixed (yellow, blue, black, or red). Secondly, additional tests comparing the behavior in the colored mark and mirror condition with that in the colored mark and no-mirror condition were carried out with a normal cage grid instead of a Plexiglas wall in front of the cage. In experiments with dolphins [9], the interpretation of the subjects' behaviors was complicated by the fact that the animals had apparently used the reflecting sidewalls of their pool in addition to the mirror provided by the experimenters. Although reflectance by the Plexiglas front was not likely, we sought additional controls to ensure that the presence or absence of the mirror was the only predictor of differences in mark-directed behavior.

While the birds were being set into or removed from the experimental cage, room lights were switched off. A test began by turning the lights on. Experiments were monitored via a video system from an adjacent room, and all tests were videotaped. From videotapes, the frequency of behaviors directed towards the own body were scored. Actions towards the marked region with the beak or the foot entered the analysis as mark-directed behavior (see Figure 2); all other behaviors towards the own body, such as touching the breast region with the beak, preening of the tail or wings, were considered not mark related. Likewise, all social behaviors, such as aggressive displays in front of the mirror, were scored. Figure 2 shows examples of the behaviors used for quantitative analysis. See also the supplementary material, Video S5–S8. Quantitative assessment of behavior based on the video tapes was carried out independently by two observers (H. P. and O. G.). Their scores were highly correlated (*r* = 0.98), and the combined score from both assessments was used for further analysis.

## Supporting Information

Nine video clips that show relevant behavioral sequences for the subjects Gerti, Goldie, Harvey, and Schatzi.

Video S1 Visual Exploration of the Mirror Image, Four Examples, Subject Gerti The bird is close to the mirror, behaves in a rather quiet way, and explores its mirror image. This behavior contrasts sharply with the agitated and sometimes aggressive behavior as observed in the subjects Harvey and Lilly (see Video S9 for examples).
(3.89 MB WMV)

Video S2 Behavior in the Preference Test, Schatzi The bird had a strong preference for the compartment with the mirror and explored the situation intensively. Note the repeated sequences of looks into the mirror and behind the mirror.
(3.98 MB WMV)

Video S3 Testing for Contingencies, Gerti Gerti is testing for contingencies between her own behavior and the mirror image by moving back and forth and left and right in front of the mirror. Note the behavior after 20 s when Gerti very slowly bends to the left and inspects her mirror image with the mark. We never observed similar behavior in any social interaction between two magpies.
(1.93 MB WMV)

Video S4 Testing for Contingencies, Goldie Goldie explores his mirror image while moving back and forth and turning his head in front of the mirror.
(2.78 MB WMV)

Video S5 Attempts to Reach the Mark with the Beak, Gerti Gerti tries to reach the mark with the beak, which is not possible. In the second of the two scenes, she finally takes her right foot and successfully removes the mark.
(3.10 MB WMV)

Video S6 Mark-Directed Behavior with the Foot, Schatzi Schatzi attempts to reach the mark with the right foot.
(674 KB WMV)

Video S7 Mark-Directed Behavior with the Foot, Gerti Three examples of mark-directed behavior with the foot by Gerti. The three examples show how the birds used their feet for mark-directed behavior. They also show that this behavior, as with mark-directed behavior with the beak, is clearly distinguishable from actions towards other parts of the body.
(2.52 MB WMV)

Video S8 Attempts to Remove the Mark, Gerti The example shows how Gerti tried to support mark removal with the beak by means of bill swipes. Note that after having removed the mark, Gerti turns to the mirror and inspects her mirror image before beginning comfort behavior.
(2.19 MB WMV)

Video S9 Aggressive Behavior, Harvey Four instances of social, in particular, aggressive behavior in the subject Harvey. In the first two scenes, the behavior shows a transition between submissive and aggressive behavior; the last two scenes represent outright attack-like behavior.
In some of the scenes filmed at an early stage of the experiments, Gerti was in a phase of molting. Later scenes show the same subject with full plumage after molting had ceased. Note that birds with lateral eyes fixate objects by viewing laterally with either the right or left eye.
(2.47 MB WMV)
